# Effect of Bleaching Agents on Composite Resins with and without Bis-GMA: An In Vitro Study

**DOI:** 10.3390/jfb15060144

**Published:** 2024-05-27

**Authors:** María Melo, Bianca Dumitrache, James Ghilotti, José Luis Sanz, Carmen Llena

**Affiliations:** Department of Stomatology, Faculty of Medicine and Dentistry, Universitat de València, Gascó Oliag, 46010 Valencia, Spain

**Keywords:** Bis-GMA, color, composite, hardness, roughness

## Abstract

The objective was to evaluate the change in color, hardness, and roughness produced by carbamide peroxide (CP) at two different concentrations on two resins. The 16% or 45% CP was applied to 66 resin discs with and without Bis-GMA. The color was measured with a spectrophotometer, and ΔE_ab_ and ΔE_00_ were calculated. Microhardness tester and SEM were used. In both composites, the a* and b* coordinates tended to be red and yellow, respectively, and were significant in the Bis-GMA group (*p* < 0.05). The ΔE_ab_ and ΔE_00_ were higher in the composite with Bis-GMA, regardless of the treatment received (*p* < 0.05). The microhardness was reduced in both composites regardless of the PC concentration compared to the control (*p* < 0.05). The 45% CP reduced the microhardness in the resin group with Bis-GMA compared to 16% CP (*p* < 0.001) but was not significant in the resin without Bis-GMA (*p* = 1). An increase in roughness was directly proportional to the concentration of CP, and it was more notable in the composite without Bis-GMA. The composite with Bis-GMA showed a greater tendency to darken than the one without Bis-GMA. The surface hardness of the composite was reduced in both composites and was not influenced by CP concentration in the composite without Bis-GMA. Bleaching is a common procedure nowadays. It is important to know how CP affects composites to establish a prognosis of the treatments in terms of color change, roughness, and hardness.

## 1. Introduction

Carbamide peroxide (CP), also known as urea peroxide, decomposes into hydrogen peroxide (HP) and urea. The bleaching action is produced by the release of reactive oxygen species (ROS) produced by HP, which exert an oxidizing effect on organic molecules by replacing carbon with colorless hydroxyl radicals (OH) [1]. This oxidative action is not only produced on the chromophores that darken the tooth but also on the organic component of dentin and composite resins used in restorative treatments, causing alterations in their structure. [2]. These alterations may vary according to different factors, such as the composition of the restorative material, the frequency and duration of exposure to the bleaching agent, and the composition and concentration of the bleaching gel. The most frequent changes that occur are the alteration of color and opacity, the increase of surface roughness, the decrease of microhardness, and the reduction of the adhesion strength of the material to the tooth [2,3].

Increased surface roughness of the resin can lead to increased plaque accumulation, which in turn can cause oral health problems, for example, at the gingival level [4]. In turn, a high surface roughness of the restorative material, together with the reduction of its microhardness, may favor surface staining of the composite [2]. This plays an important role in dental esthetics, as does resin color change. All these factors can adversely affect the longevity of the material, leading to failure of the restoration and often requiring replacement [5].

In the field of direct restoration, the current treatment of choice is the use of composites whose matrix is composed of bisphenol A (BPA) derivatives, which are liquid monomers that solidify upon polymerization, either chemically or by means of light [6]. Some of the most significant are bisphenol A glycerolate dimethacrylate (Bis-GMA) and bisphenol A ethoxylate dimethacrylate (Bis-EMA) [6], although there are also others such as bisphenol A dimethacrylate (bis-DMA), BPA diglycidylether (BADGE), triethylene glycol dimethacrylate (TEGDMA), and urethane dimethacrylate (UDMA). The latter two are used to maximize the viscosity of the resin [7]. UDMA is currently the only commercial alternative to BPA-based dental dimethacrylates [8]. Compared to Bis-GMA, UDMA has a lower molecular weight, which results in a slightly higher polymerization shrinkage. However, it has better mechanical properties due to its strong hydrogen bonds [9]. To decrease polymerization shrinkage in resins, compounds such as high molecular weight aromatic urethane dimethacrylate (AUDMA) or addition-fragmentation monomers (AFM) are sometimes added. AUDMA decreases the number of reactive groups in the resin, thus controlling volumetric shrinkage and matrix stiffness. In parallel, AFM acts by forming cross-links between adjacent polymer chains, relieving stress in the polymerization reaction. In this regard, the use of the layering technique is of great importance to minimize the stress between the tooth and the composite layer, as well as to achieve the correct reach of the light-curing light in depth [10]. Due to the frequent use of composite resins in daily practice, it is essential that they have optimal properties and that these properties are maintained over time [11].

For color determination, it is currently recommended to use the CIEDE2000 color difference formula, as it better represents the human perception of color differences (95% agreement with visual results) than the CIELAB formula (75% agreement) [12]. The most commonly used bleaching agents are HP or CP in gel form and in concentrations that vary considerably depending on the agent used [4,13].

During in-home bleaching, the concentrations of the products currently used under professional supervision are CP up to 18% or HP up to 6%, according to current European regulations [14]. For in-office bleaching, higher concentrations of PH (>6%) or PC (>18%) are used, with different application protocols [15]. Several studies have evaluated the effects of HP on restorative materials [1]. Some of them have shown that the resin matrix is easily altered by bleaching agents, causing changes in the color, microhardness, and surface roughness of the restoration [2]. However, others claim that there are only minor changes or no changes in the restoration materials [16,17]. This controversy in the results may be due, in addition to the characteristics and concentration of the active agents (HP or CP), to other elements contained in the bleaching products that may influence the final effect they exert on the substrate. The different composition of composite resins may also condition their response to the action of bleaching products in clinical practice [4].

Within this framework, the aim of this study was to evaluate the changes in the surface, hardness, and color of two composites with and without Bis-GMA matrix when subjected to the action of two bleaching agents.

The research hypotheses tested in the present study were that CP affected the color, hardness, and roughness produced in composites with and without Bis-GMA.

## 2. Materials and Methods

For the present study, two composites with the same particle size and shade (A2) were used, one with a Bis-GMA-based matrix (Filtek Supreme XTE Universal, 3M ESPE, Dental Products, St. Paul, MN, USA) and the other with a Bis-GMA-free matrix (Filtek Universal Restorative, 3M ESPE, Dental Products, St. Paul, MN, USA). As bleaching agents, two CP-based products from the same manufacturer were used at two different concentrations (Opalescence PF Regular 16% and Opalescence Quick PF 45%, Ultradent Products, Cologne, Germany). Table 1 summarizes the composition and characteristics of each of the materials and products used.

A total of 66 cylindrical samples (n = 33 in each group), 7 mm in diameter and 2 mm thick, of each composite were prepared using a disc former (Sampler, Smile Line, Brandenburg, Germany). The material was adapted to the composite using a composite spatula and flattened with a 500 g glass slab for 30 s, light-curing each disc on both sides for 40 s with the Radii-Cal lamp (SDI, Victoria, Australia) with an emitted radiation of 1200 mW/cm^2^, tested prior to use in both groups with the Proclinic expert radiometer (Proclinic, Zaragoza, Spain). The upper surface of each sample was polished for 1 min using silicon carbide sandpaper discs (Soft-Lex, 3M ESPE, Dental Products, St. Paul, MN, USA) of three different thicknesses, from the thickest to the finest grain size for 20 s each. They were then polished with felt disks with aluminum oxide paste from (Enamel Plus Shiny C, Micerium S.p.A, Avegno, Italy). The samples were stored in distilled water at 37 ± 2 °C, until use.

**Figure 1:**
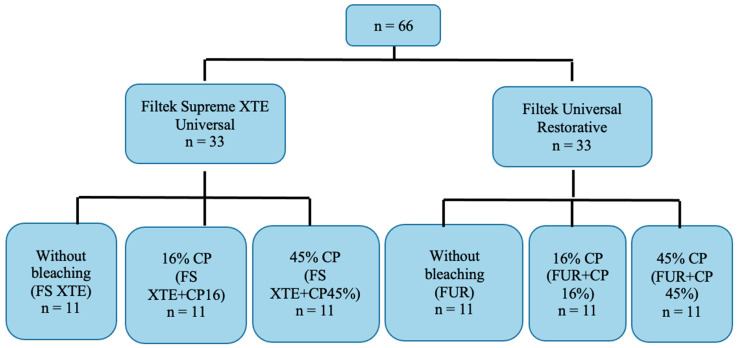
Study groups.

The 16% CP was applied for 5 h a day for 14 days in each group. For the groups treated with 45% CP, a single 30 min application was made, following the manufacturer’s recommendation. The bleaching agent was applied to the polished surface of the composite and kept at 37 ± 2 °C for 5 h or 30 min, depending on the group, thus simulating the oral cavity in a humid and dark environment. They were then washed with running water for 1 min, dried with absorbent paper, and stored again at 37 ± 2 °C in distilled water. Parallely, the untreated negative control groups were stored under the same conditions. Figure 1 shows the distribution of the study groups.

The color of the resin samples was analyzed using the VITA Easyshade V spectrophotometer (VITA, Bad Säckingen, Germany). The spectrophotometer was calibrated before starting the color measurements for each group. The color determinations were performed in a completely dark environment, and the samples were placed on a neutral gray background [18]. The chromatic parameters of the CIELab space provided by the spectrophotometer were recorded in its three coordinates: L*, a*, and b*. Two measurements were made for each of the groups, an initial one 24 h after the preparation of the samples and a final one 24 h after the end of the bleaching treatment [3].

The difference in color before/after bleaching was determined using two indices: the ΔE*_ab_, according to the following formula ΔE = [(L_a_ − L_b_)^2^ + (a_a_ − a_b_)^2^ + (ba − b_b_*)^2^]^1/2^, and the ΔE_00_ according to the following formula [2]:

$$\Delta E_{00}={\left[{\left(\frac{{\Delta L}^{\prime }}{K_{L}S_{L}}\right)}^{2}+{\left(\frac{{\Delta C}^{\prime }}{K_{C}S_{C}}\right)}^{2}+R_{T}\left(\frac{{\Delta C}^{\prime }}{K_{C}S_{C}}\right)\left(\frac{{\Delta H}^{\prime }}{K_{H}S_{H}}\right)\right]}^{1/2}$$

Two samples in each group were randomly selected. Each sample was mounted on aluminum stubs, and then the samples were gold-sputtered by the Polaron Range SC7640 Sputter Coater (Quorum Technologies Ashford, Kent, UK), and surface roughness was qualitatively evaluated using scanning electron microscopy (SEM; Hitachi S-4800 (Hitachi High-Technologies Corporation, Tokyo, Japan), as performed in previous similar studies [17]. SEM was set on 3 HV. Magnifications were ×500, ×1500, ×2500, and ×3500.

To evaluate the microhardness of the composite, three samples were randomly selected with a microhardness tester (Struers, Ballerup, Denmark) and with a Vickers indenter (500 g/15 s). Five indentations were performed on each sample, and the mean was calculated.

The data from the color analysis and surface microhardness analysis were analyzed using the statistical package SPSS Statistics Base 29.0.10 for Windows (IBM, New York, NY, USA). Descriptive data are shown graphically using the mean and standard deviation statistics. Pairwise comparisons between groups were performed using the non-parametric Mann–Whitney U test; in all cases, the significance level was 95%.

## 3. Results

### 3.1. Color Analysis

**Figure 2:**
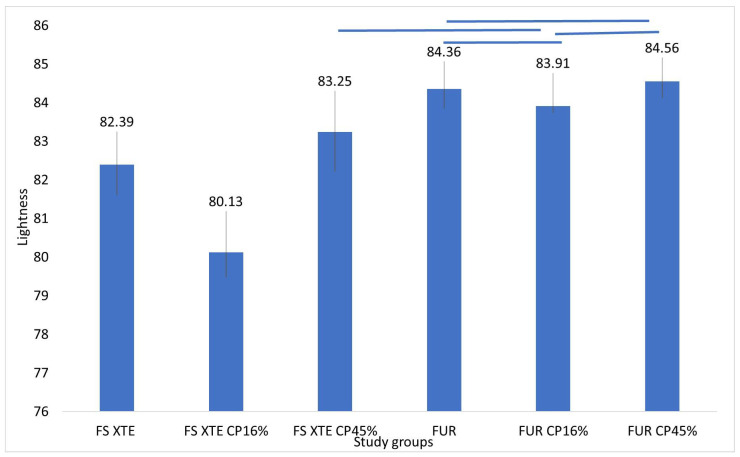
Mean values and standard deviation of lightness among the study groups. Figure legend: FS XTE: Filtek Supreme XTE Universal, FUR: Filtek Universal Restorative, CP: Carbamide Peroxide. The blue lines indicate the groups that did not show significant differences between them.

When comparing the two resins without bleaching treatment, the FS XTE resin with Bis-GMA showed a significantly lower lightness than FUR without Bis-GMA (*p* < 0.001). The FS XTE resin showed a significant reduction in lightness when treated with both 16% and 45% CP (*p* < 0.05). However, they were not significantly different in the FUR resin when they were treated with the two CP concentrations (*p* > 0.05; Figure 2).

**Figure 3:**
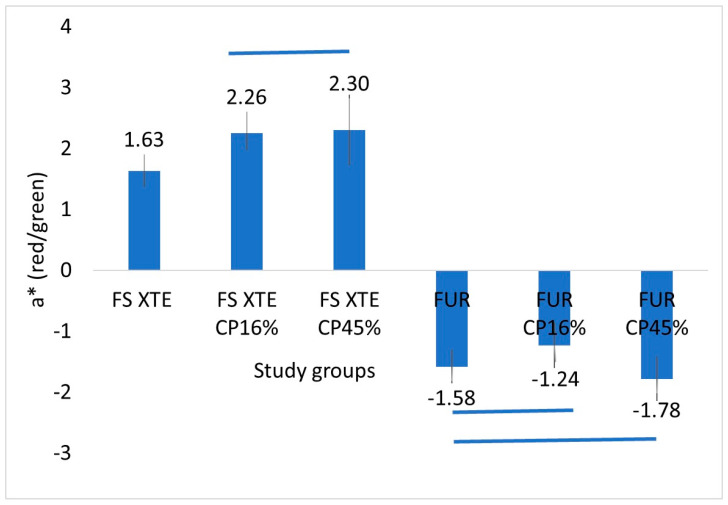
Mean values and standard deviation of the a* component (red-green distance) between the study groups. Figure legend: FS XTE: Filtek Supreme XTE Universal, FUR: Filtek Universal Restorative, CP: Carbamide Peroxide. The blue lines indicate the groups that did not show significant differences between them.

With regards to the a* component, FS XTE showed significantly higher values than FUR (*p* < 0.001), thus showing a greater tendency toward red. In the FS XTE group, both bleaching treatments increased the a* values with respect to the non-treated resin. In the FUR group, the 45% CP shifted the a* value more toward red than the 16% CP (*p* < 0.001; Figure 3).

**Figure 4:**
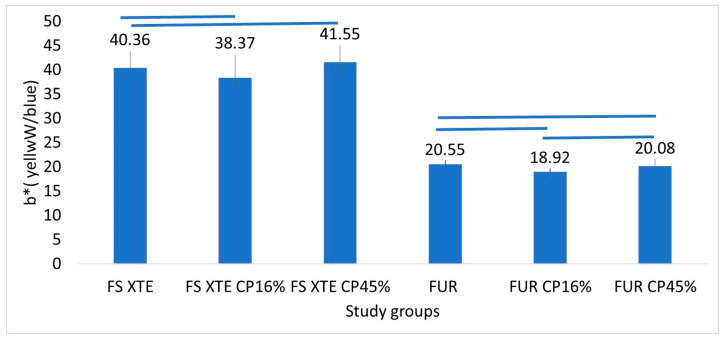
Mean values and standard deviation of the b* component (yellow-blue distance) between the study groups. Figure legend: FS XTE: Filtek Supreme XTE Universal, FUR: Filtek Universal Restorative, CP: Carbamide Peroxide. The blue lines indicate the groups that did not show significant differences between them.

All resin groups with Bis-GMA (FS XTE) showed significantly higher b* values than the resin without Bis-GMA (FUR). In the FS XTE resin, 16% CP significantly reduced the b* component compared with the 45% CP (*p* = 0.02), while no differences were found between untreated resin and resin bleached with the two concentrations of CP (*p* > 0.05). In the FUR groups, b* values showed non-significant differences between both bleaching concentrations (*p* > 0.05; Figure 4).

The ΔE_ab_ for FS XTE and FUR resins between the control group and the groups treated with 16% CP or 45% CP are shown in Table 2. No significant differences were observed between the two CP concentrations, for which *p* = 0.19 and *p* = 0.15, respectively. When PC concentrations for both resin composites where analyzed, a significantly higher change was obtained in the composite containing Bis-GMA (FS XTE) than in the one without Bis-GMA (FUR) *p* < 0.05). For ΔE_00_, the trends were the same. The values are shown in Table 2.

### 3.2. Qualitative Evaluation of Surface Roughness

**Figure 5:**
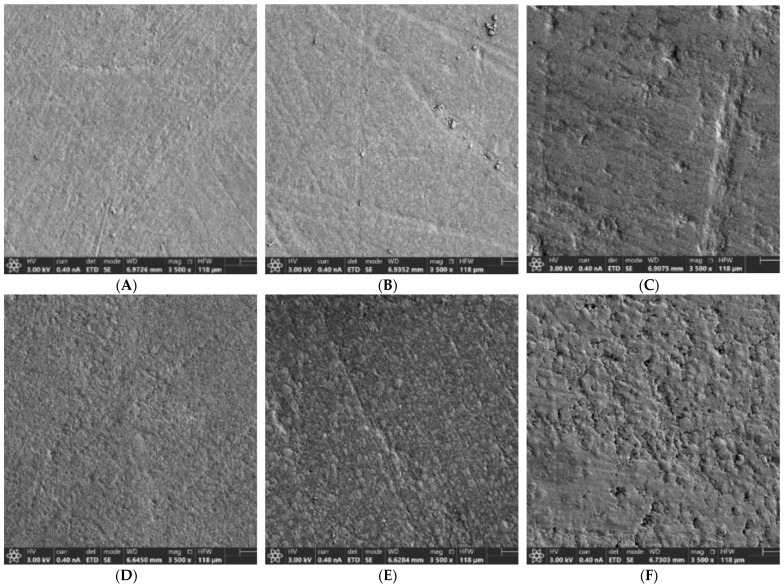
Representative SEM images of the surface of the different groups (×3500 magnification). Figure legend: (**A**): FS XTE (Filtek Supreme XTE Universal) control, (**B**): FS XTE + CP16%, (**C**): FS XTE + CP45%, (**D**): FUR (Filtek Universal Restorative) control; (**E**): FUR + CP16%, (**F**): FUR + CP45%.

SEM images showed greater roughness in the samples subjected to bleaching with regards to their controls in both groups (panels A and D; Figure 5). The Bis-GMA-treated composite samples (panels B and C; Figure 5) exhibited a more regular surface than the Bis-GMA-free composite (panels E and F; Figure 5). Within each resin, an increase in surface roughness directly proportional to the increase in the concentration of the CP used was observed.

### 3.3. Surface Microhardness Analysis

**Figure 6:**
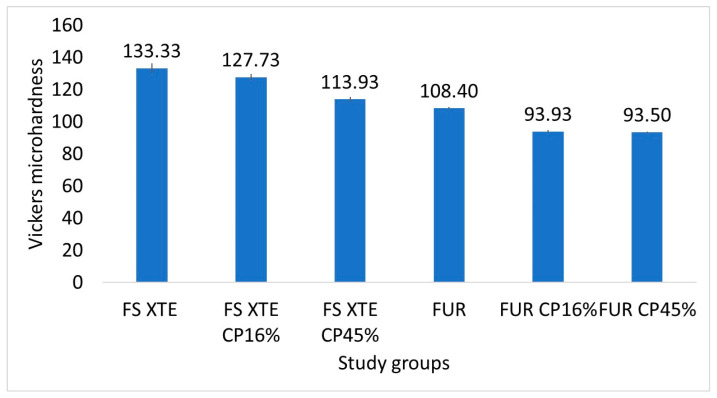
Vickers microhardness values (mean and standard deviation) by groups. Figure legend: FS XTE: Filtek Supreme XTE Universal, FUR: Filtek Universal Restorative. CP: Carbamide Peroxide.

The composite without Bis-GMA had a significantly lower microhardness (108.40 ± 0.69) compared to the composite with Bis-GMA (133.33 ± 2.80; *p* < 0.001), even without any bleaching treatment. The surface microhardness of the Bis-GMA composite group decreased significantly depending on the bleaching gel concentration (FS XTE). However, the microhardness in the composite without Bis-GMA (FUR) decreased significantly after bleaching with the two gel concentrations (*p* < 0.001). No differences were observed between the two concentrations (*p* = 1; Figure 6).

## 4. Discussion

Composite resins have a wide variety of organic compounds that can contribute to intrinsic discoloration. Bis-GMA is one of the most used monomers in composites. However, several authors claim that this component can be released during polishing and even with the degradation of dental materials. For this reason, the industry presents alternatives where other types of monomers are used to replace the conventional ones [19,20].

The differences in color observed between the composites used in this study may be due to their different chemical compositions. Both composites used are nanofilled, but with differences in their composition. In FS XTE, the fillers are a combination of non-agglomerated/non-aggregated 20 nm silica filler, non-agglomerated/non-aggregated 4 to 11 nm zirconia filler, and aggregated zirconia/silica cluster filler (comprised of 20 nm silica and 4 to 11 nm zirconia particles) in an amount of 78.5% by weight and 63.3% by volume, in a matrix of Bis-GMA, UDMA, TEGDMA, and bis-EMA. On the other hand, the FUR composite filler has, in addition to the above-mentioned components, an ytterbium trifluoride filler consisting of agglomerated 100 nm particles, in a proportion 76.5% by weight (58.4% by volume). The matrix is formed by AUDMA, AFM, diurethane-DMA, and 1,12-dodecane-DMA [21,22].

The color changes recorded in the present study after the application of 16% and 45% CP were in the moderately acceptable range of (50:50% AT; AT: ΔΕ_ab_* < 5.4 and ΔΕ_00_ < 3.6), although it was in the range of perceptible (50: 50% PT) for the composite with Bis-GMA (PT: ΔΕ_ab_* < 2.7 and ΔΕ_00_ < 1.8). This means that although there were color changes in both composites, they would presumably be acceptable by standard observers [12]. These results coincide with those obtained by Karanasiou et al. [23], where, although the color changes were in the acceptable range, a greater color change was found after bleaching treatment in the composite with Bis-GMA matrix. Similar results were found by Mendes et al., who evaluated the effect of HP at 10% and 35% on the surface of a nanohybrid composite and another one with nanoparticles, both with Bis-GMA and different particle sizes [24]. In the present study, it is important to highlight that the FUR composite contains less filler both in weight and in percentage compared to FS XTE, which may influence the difference in the values of the chromatic coordinates in the material of the same color (A2 according to the Vita Classical guide). In another study carried out by Vilalta et al. [25], the color change of the composite was related to intrinsic factors of the composite, such as the chemical composition of the material, quality and quantity of organic matrix, type and quantity of inorganic filler, photoinitiating agent used, and degree of monomer conversion. The conclusion of this study was that, after applying stains and then bleaching the composite resins, the composite resins became lighter due to the removal of extrinsic stains, but there was no intrinsic bleaching effect. Different authors also state that the type and concentration of the bleaching agent determine the response of the composite [26,27].

According to the literature, the surface of the resin with Bis-GMA has a more regular surface with lower roughness than the resin without Bis-GMA, and the roughness increases with the use of bleaching agents [28,29]. In the present study, in both composites, the surface of the samples exhibited a greater roughness with 45% CP treatment, coinciding with other published studies [30]. High energy free radicals from bleaching agents can easily affect the organic matrix of the composite, softening the material and disrupting the resin-filler interface. As a consequence, disconnection of the filler occurs, leaving a larger crater that will result in increased surface roughness [31]. In the present study, the FUR group, which contains a lower amount of filler both in weight and in percentage compared to FS XTE, showed a higher surface roughness. This lower filler load may have a negative influence on the surface roughness when the composite is affected by a bleaching agent. Likewise, even though they are nanofilled composites, the particle size is larger in FUR than in FS XTE, which may also influence the higher surface roughness in this material.

According to several authors, the changes in the surface of the material are due to differences in the matrix composition of the composites [4,32] together with the bleaching product itself and the duration of the bleaching treatment [33]. In the study by Yikilgan et al. [34], after analyzing the roughness produced after the application of bleaching agents of different concentrations, they observed that none of the bleaching agents used showed significant effects on the surface roughness of the composite resin. The average roughness was 0.2 µm, which was considered clinically acceptable. Fernandes et al. [4] also stated that bleaching agents have no effect on the surface roughness of composite resins or only have an effect in some cases. These results do not agree with those obtained in the present study, where an increase in roughness was observed after the use of the bleaching agent. This was also observed in the study by Al-Angari et al. [31], who observed an increase in surface roughness due to the bleaching treatment in the three substrates (dental enamel, nanohybrid resin, and microhybrid resin). The microhybrid composite was the most affected. Roughness on the surface of the materials increases the retention of bacterial plaque, which has a negative effect on the stability of the material, as well as on the response of the gingival tissue [35].

The composition of composites is directly related to their hardness. Bis-GMA has the highest hardness, followed by TEGDMA and, finally, UDMA. Furthermore, the addition of UDMA to the composition of Bis-GMA and TEGDMA exponentially increases the hardness of the copolymer [8]. These results coincide with those obtained in our study, in which the FS XTE group showed significantly higher values than the FUR group, regardless of whether they were treated or not with CP and its concentration. However, there is no agreement in the literature when it comes to determining the effect of bleaching agents on the hardness of composites. While some authors affirm that there are no differences in the tests before and after application, others point out that the hardness decreases [36], as was the case in the present study, while in the Türker and Biskin study [17], the use of 16% CP increased the composite’s hardness. In AlQahtani’s study, surface hardness was found to be affected in mainly silorane-based matrix composites, which were softer than others based on Bis-GMA or UDMA, and more easily soluble by bleaching agents [37].

## 5. Conclusions

Within the limitations of this study, the composite without Bis-GMA (FUR) presented chromatic coordinates of greater clarity, with greater luminosity and lower values of a* and b*. The composite with Bis-GMA showed a greater tendency to darken than the one without Bis-GMA, at the expense of a greater tendency to become red (an increase of a*) and yellow (an increase of b*), within the margins of 50:50 acceptability. The composite without Bis-GMA (FUR) suffered greater surface degradation due to the action of CP. The surface hardness was reduced in both composites and was not influenced by the concentration of CP in the composite without Bis-GMA. All research hypotheses were accepted.

## Figures and Tables

**Table 1:** Materials and products used.

MATERIAL	COMPOSITION	MANUFACTURER	BATCH
Opalescence PF Regular 16% (PC16%)	16% CP (≈5.8% H_2_O_2_), glycerin, water, carbomer, PEG-6, sodium hydroxide, EDTA, potassium nitrate, sodium fluoride, xylitol	Ultradent Products, Cologne, Germany	BMCCJ
Opalescence Quick PF 45% (PC45%)	PC al 45% (≈15% H_2_O_2_), water, potassium nitrate, fluoride	Ultradent Products, Cologne, Germany	BKPPS
Filtek Supreme XTE Universal, shade A2 body (FS XTE)	Silica, zirconium, Bis-GMA, UDMA, TEGDMA, PEDGMA, bis-EMA	3M ESPE, Dental Products, St. Paul, MN, USA	4911A2D
Filtek Universal Restorative, shade A2 (FUR)	Silica, zirconium, ytterbium trifluoride, AUDMA, AFM, diurethane-DMA, 1,12-dodecane-DMA	3M ESPE, Dental Products, St. Paul, MN, USA	6555A2

**Table 2:** Mena ± SD ΔE_ab_ and ΔE_00_.

ΔEab	Mean ± SD	*p*
FS XTE/FS XTE CP16%	3.65 ± 1.96	0.19
FS XTE/FS XTE CP45%	2.79 ± 1.08	
FUR/FUR CP16%	1.93 ± 0.89	0.15
FUR/FUR CP45%	1.40 ± 0.85	
FS XTE CP16%/FUR CP16%		0.03
FS XTE CP45%/FUR CP45%		0.01
ΔE_00_		
FS XTE/FS XTE CP16%	1.97 ± 0.96	0.23
FS XTE/FS XTE CP45%	1.49 ± 0.45	
FUR/FUR CP16%	1.19 ± 0.44	0.46
FUR/FUR CP45%	0.89 ± 0.46	
FS XTE CP16%/FUR CP16%		0.02
FS XTE CP45%/FUR CP45%		0.01

FS XTE: Filtek Supreme XTE Universal, FUR: Filtek Universal Restorative. CP: Carbamide Peroxide.

## Data Availability

The data presented in this study are available on request from the corresponding author.
